# Enhancing Security on Touch-Screen Sensors with Augmented Handwritten Signatures

**DOI:** 10.3390/s20030933

**Published:** 2020-02-10

**Authors:** Majd Abazid, Nesma Houmani, Sonia Garcia-Salicetti

**Affiliations:** SAMOVAR, Telecom SudParis, Institut Polytechnique de Paris, 9 rue Charles Fourier, 91011 Evry, France; majd.abazid@telecom-sudparis.eu (M.A.); sonia.garcia@telecom-sudparis.eu (S.G.-S.)

**Keywords:** automatic signature verification, touch-screen sensor, data quality, enrollment phase, performance assessment, augmented signature, security enhancement, mobile conditions

## Abstract

We aim at enhancing personal identity security on mobile touch-screen sensors by augmenting handwritten signatures with specific additional information at the enrollment phase. Our former works on several available and private data sets acquired on different sensors demonstrated that there are different categories of signatures that emerge automatically with clustering techniques, based on an entropy-based data quality measure. The behavior of such categories is totally different when confronted to automatic verification systems in terms of vulnerability to attacks. In this paper, we propose a novel and original strategy to reinforce identity security by enhancing signature resistance to attacks, assessed per signature category, both in terms of data quality and verification performance. This strategy operates upstream from the verification system, at the sensor level, by enriching the information content of signatures with personal handwritten inputs of different types. We study this strategy on different signature types of 74 users, acquired in uncontrolled mobile conditions on a largely deployed mobile touch-screen sensor. Our analysis per writer category revealed that adding alphanumeric (date) and handwriting (place) information to the usual signature is the most powerful augmented signature type in terms of verification performance. The relative improvement for all user categories is of at least 93% compared to the usual signature.

## 1. Introduction

The handwritten signature has been for a long time a usual mean to establish personal consent, with legal value for administrative and financial institutions. With the impressive proliferation of mobile devices having embedded sensors (smartphones, tablets), added to the development of online services, signing on digital platforms has become a reality in different sectors for identity security (banking, legal transactions, e-commerce among other). This reality has signified a turning point in the field of online signature biometrics.

In the last forty years, research studies were focused on online signatures captured on high quality sensors such as Wacom digitizing tablets, in controlled office-like scenarios, with a devoted ad-hoc pen stylus. Impedovo and Pirlo [1] published an article giving a detailed overview on the state-of-the-art techniques. Diaz et al. [2] presented a recent update on automatic signature verification (ASV). The research community made significant efforts for acquiring several online signature corpora [1,2,3,4,5,6,7,8,9,10,11] and conducting international evaluations of ASV systems [2,6,12,13,14,15,16]. 

Recent research studies have focused on signature verification in mobile conditions, using touch-screen sensors largely deployed nowadays. Nevertheless, the mobile scenario implies much more variability of acquisition conditions, like posture, writing tool (stylus or finger), screen size, sensor technology, interoperability, setting several new challenging issues that impact verification performance [2,17].

Usually, for improving verification performance, different strategies were exploited in the literature: (i) acquiring signatures in controlled conditions [1,2,3,4,5,6,7,8,9,10,11,12,13,14,15,16]; (ii) using a high quality sensor (such as a Wacom tablet) with high temporal and spatial resolution, and able to capture other time functions than pen coordinates, as pen pressure and pen inclination angles [18]; (iii) selecting reference signatures in order to control intra-personal variability [19,20,21]; (iv) extracting several features for signature description (as pressure, speed, and acceleration, etc.) [1,2,3,4,5,6,7,8,9,10,11,12,13,14,15,16] or by means of a deep neural network [2,22,23,24,25].

However, some of these strategies are no longer possible in the mobile scenario: as pointed out by [17], the sensors are not of the same quality, in terms of temporal resolution in particular, acquisition conditions are highly variable, and some sensors are limited to the capture of only pen coordinates. In the so-called “cloud scenario” [17], users acquire their signatures as they want, standing, sitting or moving, handling the device on the hand at different angles or orientations, or placing it on any support. A smartphone is usually handheld, while a tablet may be placed on the desktop or sustained by the left arm if the writer is right-handed. The consequence is that verification performance is strongly degraded in mobile conditions [2,15,16,26,27,28,29,30,31,32,33,34,35,36,37,38,39]. 

In the present paper, we study the online signature biometrics in the framework of uncontrolled mobile conditions. The challenging question then is how to improve verification performance in uncontrolled mobile conditions? To respond to this question, we propose a novel and original scheme for enhancing signature information content at the enrollment phase and reinforce its resistance to attacks, on a largely deployed touch-screen sensor technology. To this end, we propose different enrollment strategies for signature enrichment and assess them in terms of data quality and verification system performance. 

The enrollment phase is critical for any biometric system since it determines the genuine signatures that will represent the user at the verification step. These signatures are called “Reference signatures”. In our previous works on signature quality assessment, we have shown that a signature’s resistance to attacks depends on its information content, quantified by an entropy-based measure, called personal entropy [28,40,41,42,43]. We identified automatically different risk levels in signatures related to three user categories, and in particular a “problematic” population, characterized by simple and highly variable signatures, very vulnerable to attacks.

Based on these findings, we propose in this paper, since the enrollment phase on a touch screen sensor, a novel strategy that turns any signature with a “high risk” into a “low risk” one. For signature enrichment, we use complementary personal handwritten information, as initials, name-surname, date and place of birth. We choose these information since a person is familiarized to append it for expressing her consent in administrative or legal frameworks. For this study, we consider different types of signatures (the usual signature, initials, name-surname, date and place of birth) and hybrid types as well (some combinations of the already mentioned types), and analyze the impact of each in terms of information content and resistance to attacks (skilled forgeries). 

This paper is organized as follows: in Section 2, we present previous works of the literature related to online signature analysis on mobile devices. In Section 3, we describe the signature database and recall the personal entropy concept and the verification system used. In Section 4, we report the obtained results later summarized and discussed in Section 5. Finally, Section 6 presents the conclusions and future perspectives of our study.

## 2. Related Works 

Different works of the literature pointed out the diversity of acquisition conditions and the subsequent degradation of verification performance in the mobile context [2,15,16,26,27,28,29,30,31,32,33,34,35,36,37,38,39]. The majority of these works focused on assessing and improving verification performance in several ways. 

Martinez-Diaz et al. [26] indicated that ASV systems traditionally used on signatures acquired with Wacom digitizing tablets in an office-like scenario should be adapted in the context of handheld touch-screen devices. Indeed, the Wacom digitizing tablet used needs to be connected to a personal computer and thus leads to an office-like scenario. The authors exploited the BioSecure datasets DS2 and DS3 that contain signatures of the same 120 persons acquired, respectively, on a Wacom digitizing tablet following an office-like scenario and on a mobile touch-screen sensor (PDA) while holding it in the hand. Based on a feature selection algorithm and a hidden markov model (HMM) classifier, they observed the low discriminative power of dynamic features and the high consistency of geometric features in the mobile scenario.

Houmani et al. [27] evaluated an HMM-based classifier on two different databases both acquired on a PDA, namely PDA-64 containing online signatures of 64 persons, and BioSecure DS3 dataset (DS3-210) containing online signatures of 210 persons. Experiments showed significant performance degradation in mobile conditions: an average Equal Error Rate (EER) of 3.5% is obtained on the Wacom digitizer with skilled forgeries, while on PDA-64 and DS3-210, the EER is of 16.02% and 9.95% respectively. 

In the context of the international online signature competition BSEC’2009 [15], different ASV systems were assessed on two large BioSecure datasets containing signatures of the same 382 persons acquired in a controlled scenario on a Wacom digitizer, namely DS2-382, and on a mobile device (PDA), namely DS3-382. Results showed a clear degradation of systems’ performance when signing in mobile conditions. 

Another work on BioSecure databases was conducted by Houmani and Garcia-Salicetti [28] to quantify the quality of signatures of the same 104 persons when captured in office-like conditions (DS2-104, captured on a Wacom digitizer) and in the mobile context (DS3-104, captured on a PDA). Results showed that signatures’ quality degrades in mobile conditions and especially signature complexity decreases.

Blanco-Gonzalo et al. [29] evaluated an ASV system based on dynamic time warping (DTW), on seven mobile devices (using stylus and finger): two Wacom tablets, a tactile laptop, a Samsung Galaxy Note, an iPad, a Samsung Galaxy Tab and a Blackberry Playbook. They confronted the system with different acquisition conditions, such as sensor technology, interoperability, visual feedback, and screen size. Experiments showed that the best system performance is obtained when signing on a small screen, using a stylus instead of the finger. However, this study is of limited scope because only 11 writers were considered. In [31], Blanco-Gonzalo et al. exploited a DTW-based classifier on a database containing signatures of 43 users acquired on different mobile platforms. However, performance assessment was carried out only on random forgeries. 

Martinez-Diaz et al. [32] presented the first publicly available database collected on a touch-screen sensor embedded in a mobile phone, namely the DooDB database. The database contains finger-drawn doodles and pseudo-signatures from 100 persons and skilled forgeries for all of them. To create pseudo-signatures, participants were asked to draw a simplified version of their signature, for example signing with their initials or part of their signature flourish, which could be used as a graphical password. Using a DTW-based classifier, the authors obtained an EER of around 26.9% on skilled forgeries considering time variability and 19.8% when the system was evaluated on only one session.

Sae-Bae and Memon [34] collected a new database that contains finger-drawn signatures from 180 persons captured in uncontrolled mobile conditions, on user owned iOS mobile devices. The authors proposed a histogram-based feature set for representing an online signature. They pointed out the importance of updating reference signatures to reduce the intra-class variability and thus improve systems’ performance. The authors claimed that personalized feature selection is necessary to attain an acceptable performance level; they obtained an EER of 3.18% on random forgeries. 

Antal et al. [36] introduced the MOBISIG database that contains finger-drawn pseudo-signatures from 83 persons, captured on a capacitive touch-screen sensor embedded in a mobile device. Participants were asked to create a signature for a given family name and were instructed on how to produce signatures with their finger. For performance assessment, the authors used a personalized threshold; they obtained an EER of 8.56% with a DTW classifier (vs. 25.45% with a global threshold), considering skilled forgeries and five reference signatures in the enrollment phase. Then, when considering 15 samples for enrollment, they improved verification performance, reaching an EER of 5.81% with a DTW classifier and a personalized threshold (vs. 20.82% with a global threshold). 

Tolosana et al. [37] showed that performance is much better with the stylus than with the finger, on 65 persons of the e-BioSign database, using signatures captured with both Wacom tablets and Samsung mobile devices. Based on a DTW classifier, the authors obtained an EER of 22.1% with the finger versus roughly 7.9% with the stylus, considering skilled forgeries. This performance is obtained on 35 persons of the evaluation dataset; the 30 remaining persons were used as a development dataset to select 23 relevant parameters among the whole set of 117 features.

Zareen and Jabin [38] used a publicly available database [33] that contains 500 signatures from 25 persons, acquired on a Samsung Galaxy Note. As no skilled forgeries were acquired, the verification system based on a feed-forward multilayer neural network was evaluated only on random forgeries. The authors obtained an EER of 0.12% on random forgeries. These results seem preliminary since only 25 persons were considered and no skilled forgeries were used for performance assessment.

Nam et al. [39] used a private database that contains real finger-drawn signatures of only 20 persons, collected on a Samsung Galaxy S3. The authors proposed convolutional neural networks (CNN) for feature extraction, trained with genuine and forged signatures. Then, using an autoencoder for classification, they obtained an EER of 4.4% on skilled forgeries. However, this study is of limited scope because only 20 writers were considered. 

All the above-mentioned works pointed out the degradation of systems’ performance in the mobile context. However, in most of them, the data corpora presented do not contain signatures acquired in totally uncontrolled mobile conditions. This specific point is sometimes not even mentioned in the description of the acquisition protocol used, mainly focused on the sensor characteristics (technology, resolution, sampling rate), the writing tool, the design of the interface for acquisition, and the number of captured genuine signatures and forgeries. In addition, some works [32,36] evaluated the impact of mobile conditions based on pseudo-signatures, which are not exploited in real-world usages. Other studies evaluated ASV systems only on random forgeries because of the burden of acquiring skilled forgeries [34,38]. Finally, some works considered new challenging scenarios for ASV system assessment in terms of interoperability, as enrolling the person with a given writing tool and testing the system with another one [37]. 

In conclusion, we notice significant efforts in the literature for assessing verification performance in mobile conditions with different sensors, scenarios and classification strategies. Most works focused on the development of algorithms for biometric verification to enhance user authentication: DTW, HMM, neural networks and more recently some deep architectures. However, none of these works addressed quality-driven signature verification since the enrollment step, by quantifying and enhancing the information content of input data given to the sensor.

## 3. Materials and Methods

In this section, we first describe the signature database acquired for this study in mobile conditions and the signature types considered for enhancing the information content of the data given as input to the sensor. Then, we recall how Personal Entropy is quantified considering each signature type, and the classifier used for assessing the impact of our strategy in terms of verification performance.

### 3.1. Signature Data Acquisition

For this study, we captured online signatures from 74 persons on an iPad tablet with a capacitive touch-screen of 2048 × 1536 pixels. The signatures were sampled at 63 Hz and stored as a sequence of discrete values $\left[x_{t},\;y_{t}\right]$, where $x_{t}$ and $y_{t}$ are the coordinate values and *t* is the time stamp. 

Each person signed 25 times with their usual signatures. No instructions were given to participants when they signed, letting them acquire their signatures naturally, freely in terms of posture and position of the device, so that they would feel comfortable with the mobile device when signing. This leads to different acquisition conditions according to persons, exactly like it would be in real mobile usages.

Additionally to their usual signatures, we asked participants to append other types of signatures separately: name-surname, initials, date and place of birth. We considered these signature types because: (i) in terms of usages, they are traditionally reported by persons in legal and administrative documents; (ii) they convey complementary handwritten information on the user’s identity. Each type of signature was done by the person 25 times. This dataset thus contains 9250 (74 × 25 × 5) genuine signatures of different types.

**Figure 1:**
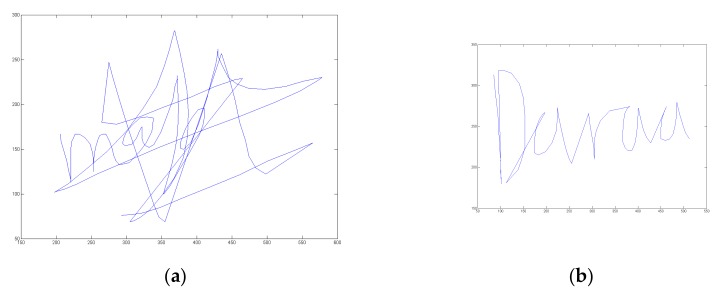
Examples of (**a**) a usual signature and (**b**) the associated place information of a user who authorized their publication.

**Figure 2:**
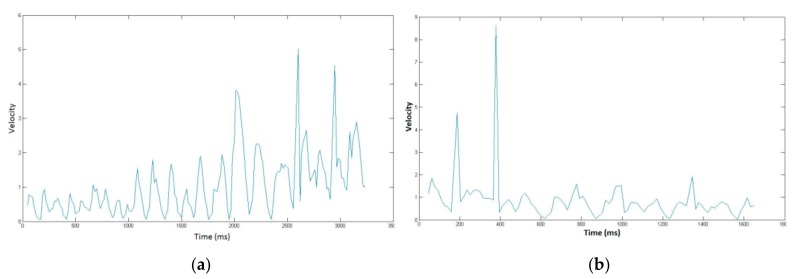
Velocity profile of (**a**) the usual signature and (**b**) the associated place information displayed in Figure 1.

Figure 1 displays an example of one person’s usual signature and the associated place of birth. We plot below in Figure 2 the velocity temporal function for both handwritten information. 

**Figure 3:**
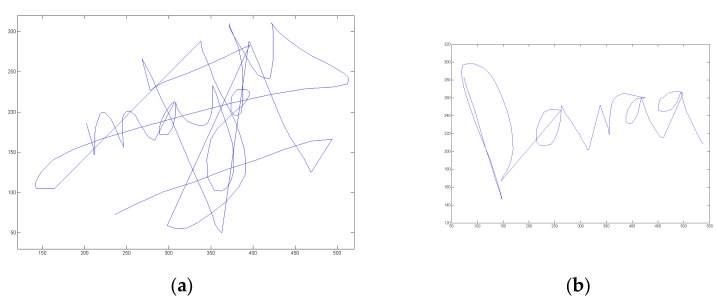
Examples of skilled forgeries of (**a**) the usual signature and (**b**) the associated place information displayed in Figure 1.

**Figure 4:**
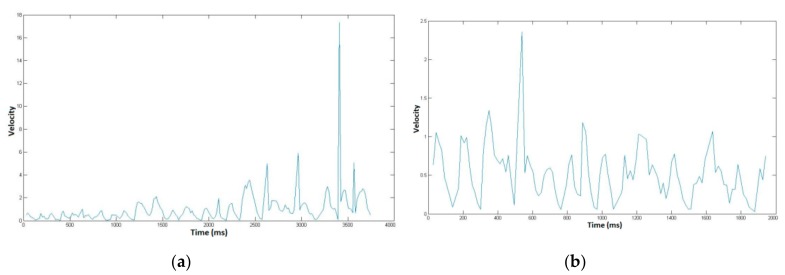
Velocity profile of (**a**) the forged usual signature and (**b**) the forged place information displayed in Figure 3.

In order to assess signature vulnerability to attacks, we acquired 10 skilled forgeries per signature type after displaying on the screen the shape and kinematics of the target signature. This type of forgery is considered in the literature as being the best attacks [3,43,44]. We thus obtain 3700 skilled forgeries (74 × 10 × 5) done by different forgers. Figure 3 shows an example of skilled forgeries of the usual signature and the associated place of birth displayed in Figure 1. We also display in Figure 4 the velocity temporal function for both handwritten information forgeries.

### 3.2. Signature Types

We considered the five types of signatures separately: the usual signature (S), the initials (I), the name-surname (N), the date of birth (D), and the place of birth (P). From these five simple types, we constructed 7 hybrid signature types by combining:

- the usual signature with initials (SI);
- the usual signature with name-surname (SN);
- the usual signature with date (SD);
- the usual signature with place (SP);
- the usual signature with date and place (SDP);
- the usual signature with initials, date and place (SIDP);
- the name-surname with date and place (NDP).

These instances of hybrid types were constructed by concatenating the sequences of the corresponding simple signature types, resulting in a single time sequence. The identity of the user is thus expressed through several signature types of different length, which convey different complementary information to strengthen the user’s identity.

### 3.3. Quantifying Quality of Signature Types

To assess information enrichment at the enrollment phase, we quantify the information content of other simple signature types than the usual signature (initials, name-surname, date and place), and also of the 7 hybrid types mentioned in Section 3.2.

The concept of entropy is a good alternative for quantifying the information content or the disorder in signatures. In [28,40,41,42,43], we proposed the concept of personal entropy (PE), an entropy-based quality measure that quantifies simultaneously both the complexity and variability of a person’s signatures. In fact, complexity and variability are related to disorder at two different levels: complexity corresponds to the intrinsic disorder in a signature sample; variability corresponds to the intra-class disorder in a set of signatures belonging to a given user.

**Figure 5:**
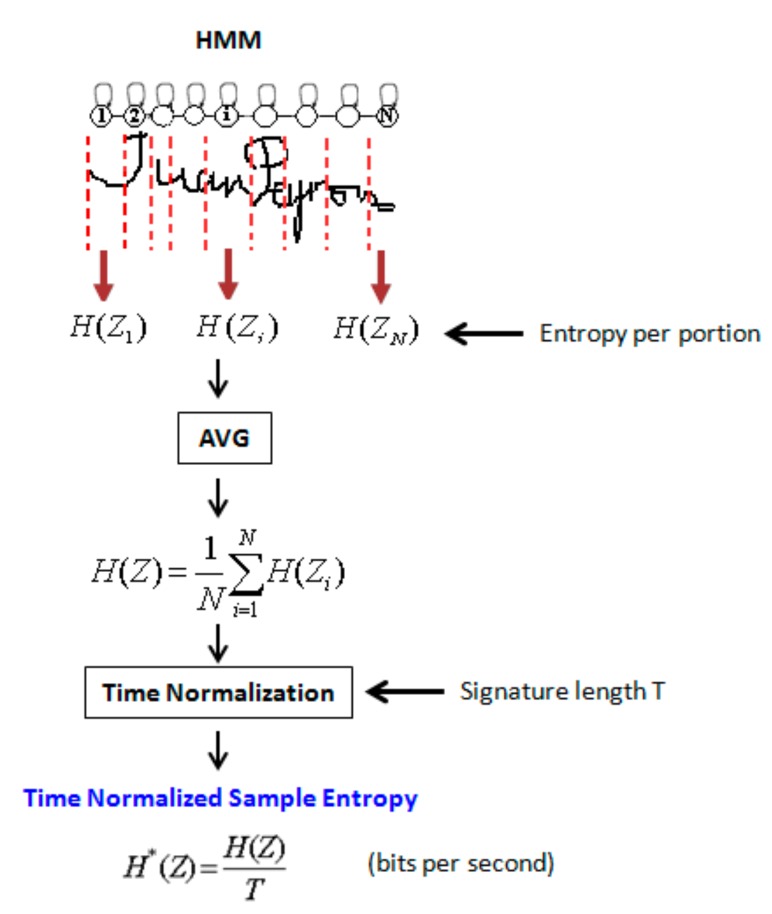
Personal entropy computation on portions of a signature.

A user’s PE is measured by exploiting the local probability densities estimated when training the user’s HMM on a set of 10 genuine signatures described only by *x* and *y* attributes. Indeed, the HMM automatically generates portions by the Viterbi algorithm and estimates a mixture of Gaussian densities on each portion [28]. Figure 5 illustrates how PE is computed locally, on the segments generated by the user’s HMM. 

Therefore, a random variable *Z* can be associated to each stationary portion *i* of the signature, generated by the Viterbi algorithm by the user’s HMM. The number of portions *N* is the number of states of the HMM. The entropy $H\left(Z_{i}\right)$ of a portion *i* is computed as follows:

(1) $$H\left(Z_{i}\right)=-\underset{z\in S_{i}}{\sum }p\left(z\right).log_{2}\left(p\left(z\right)\right),$$

where z corresponds to a given point in the signature described by its coordinates (*x,y*), belonging to the current portion *i*, and $p\left(z\right)$ is the probability of observing z. 

We studied the number of genuine samples necessary for a good HMM estimation and showed that 10 instances lead to stable PE values [28]. The local probability distribution function is estimated using all the sample points belonging to each portion, across the 10 genuine samples. After that, the entropy of each genuine signature $H^{*}\left(Z\right)$ is the average of entropy values $H\left(Z_{i}\right)$ on all the *N* portions of the signature, divided by the signing time *T*:

(2) $$H^{*}\left(Z\right)=\frac{1}{N*T}\overset{N}{\underset{i=1}{\sum }}H\left(Z_{i}\right)\;,$$

Finally, by averaging $H^{*}\left(Z\right)$ across the 10 user’s genuine samples, we obtain a user’s PE for each signature type. We demonstrated that PE allows obtaining three categories of signatures, coherent across several databases, spanning from short and highly variable signatures (high PE category) to stable, longer and complex signatures (low PE category). Moreover, we showed that for different classifiers, persons with low PE are the most robust to skilled forgeries. Persons with high PE are considered being “problematic” users in the literature [28,43,45]. These results were obtained considering the usual signature of each person [28,40,41,42,43].

### 3.4. Signature Verification System

As our aim is to assess the impact of our strategy in a mobile scenario, we used a statistical verification system that has already been evaluated on large databases acquired on mobile sensors [9,12,15,16,27,46], and has shown to maintain good performance on well-known databases in interoperability scenarios [47], as reported in Table 1. Indeed, Table 1 presents our system’s performance on several online signature databases, some acquired in an office-like scenario using a Wacom digitizer with an inking pen, and other in a mobile scenario on different touch-screen sensors (PDA, iPad, iPhone). We report the EER values on skilled forgeries only, since it is the most challenging configuration for signature verification. The system has been evaluated in BSEC’2009 and ESRA’2011 competitions on very large databases of 382 persons [15,16] that signed both on a Wacom digitizer and on a PDA device. We observe that in the mobile context, the verification performance of our system is clearly better on recent capacitive touch-screen sensors (iPad and iPhone) compared to the results obtained on the PDA device (DS3-210, PDA-64, DS3-382). 

Table 2 summarizes the state-of-the-art of online signature verification systems on mobile sensors, when considering skilled forgeries. We observe that in some publications, performance is not reported on skilled forgeries, which is the most challenging case for ASV systems. When comparing the results on mobile sensors in Table 1 and Table 2, we note that our system shows good performance compared to the state-of-the art. Indeed, on iPad and iPhone mobile sensors, an EER of 7.04% and 4.95% respectively is reached on signatures of the same 74 users. Compared to e-Biosign database containing real signatures of 65 users acquired with a stylus on two mobile devices, we notice that our HMM-based system shows slightly better performance on the iPad device (7.04% vs. 7.9% in the best case, or vs. 10.7% on the other mobile device) and much better performance on the iPhone device (4.95% vs. 7.9% in the best case, or vs. 10.7% on the other mobile device).

Our system behaves well in mobile conditions because it is based on a statistical model, namely a continuous left-to-right HMM with four Gaussian components per state [47,48,49]. In other words, each writer’s signature is modeled through a double stochastic process, characterized by a given number of states with an associated set of transition probabilities among them, and in each state, a continuous density, a multivariate Gaussian mixture is used to model the emission probability density. This model has the advantage of absorbing the intra-personal variability of signatures [47], which increases significantly in mobile conditions.

A personalized number of states is determined according to the total number $T_{total\;}$ of sampled points available in the genuine signatures of the HMM’s training set. We consider that in average 30 sampled points are enough to estimate the mean vector and the covariance matrix of each Gaussian [47]. The number of states *N* is computed as:

(3) $$N=\left[\frac{T_{total}}{M*30}\right]\;,$$

where *M* = 4 is the number of Gaussian densities per state and brackets denote the integer part.

**Figure 6:**
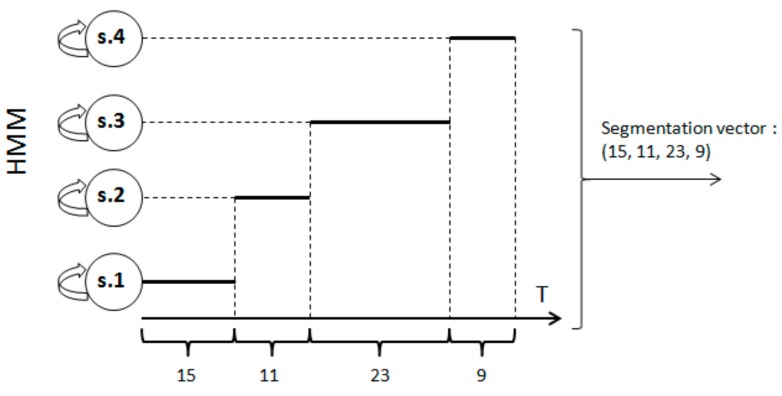
Computation of a signature’s segmentation vector generated by the user’s HMM. Feature vectors describing the signature are on the *x*-axis and the left-to-right HMM on the *y*-axis.

Nineteen dynamic features are extracted point-wise for all signature types. These features are described in detail in the Appendix A. The usual information extracted from an HMM is the likelihood of the input signature given the user’s model. We have noticed that the information coming from the segmentation of the test signature by the target user’s model is complementary to that of the likelihood, especially for forgery detection. Indeed, we have shown in [47] that the segmentations made by the target model on forgeries differ from those obtained on genuine signatures. For this reason, in the verification phase, the classifier performs a score fusion combining two levels of signature analysis: one based on a local point-wise analysis of each signature by the HMM (log-Likelihood score), the other on the analysis of the signature at the level of portions, automatically segmented by the same HMM (Viterbi score) [47,48,49]. At the first level (log-Likelihood score), on a particular test signature, a distance is computed between its log-Likelihood and the average log-Likelihood obtained on the training signatures; then it is shifted to a similarity value—called “Log-Likelihood score”—between 0 and 1, by the use of an exponential function [47]. At the second level of analysis (Viterbi score), the user’s HMM automatically performs by the Viterbi algorithm, a segmentation of each training signature into portions, according to the most likely path displayed in Figure 6. A “segmentation vector” can then be associated to each signature: the *N*-components segmentation vector, *N* being the number of states in the claimed identity’s HMM has in the *i*-th position the number of points (observations) associated to state *i* by the Viterbi path, as illustrated in Figure 6. Each training signature is then characterized by a *Reference segmentation vector*. In the verification phase, on a particular test signature, a distance between its corresponding segmentation vector and each *Reference segmentation vector* is computed, and such distances are averaged to compute the final distance. It is then shifted to a similarity measure between 0 and 1 (Viterbi score) by an exponential function [47]. 

Finally, the similarity score for a given test signature is thus the fusion by a simple arithmetic mean of the log-Likelihood score and the Viterbi score. If the final score is higher than the value of the decision threshold the claimed identity is accepted, otherwise it is rejected.

In this work, for simple signature types, we train an HMM per person and per signature type. For hybrid types, we train an HMM for each person considering the whole time sequence constructed by concatenating the time sequences of the concerned simple types. As example, for the SDP type, we train an HMM on the complete sequence composed of the usual signature, the date and the place. Note that according to signature types, the length of the complete signature sequence will vary: for simple signature types, it will tend to increase when considering name-surname and to decrease when considering initials. For hybrid types, the length of the signature will be even higher. This will impact the number of states of the user’s HMM. 

## 4. Results

### 4.1. Quality Measure of Usual Signatures

**Figure 7:**
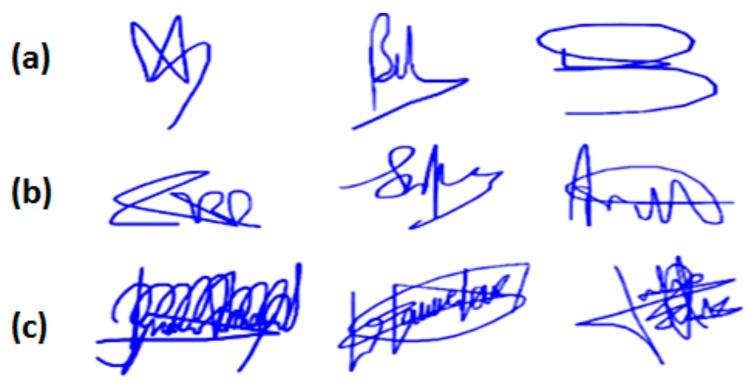
Examples of signatures captured in uncontrolled mobile conditions with (**a**) high, (**b**) medium and (**c**) low PE.

In a first step, we quantify the quality of usual signatures of the 74 persons available in our dataset. To this end, we trained for each person, an HMM on 10 genuine signatures to measure the user’s PE. Then, a hierarchical clustering was performed on the obtained PE values, resulting in three user categories displayed in Figure 7. 

**Figure 8:**
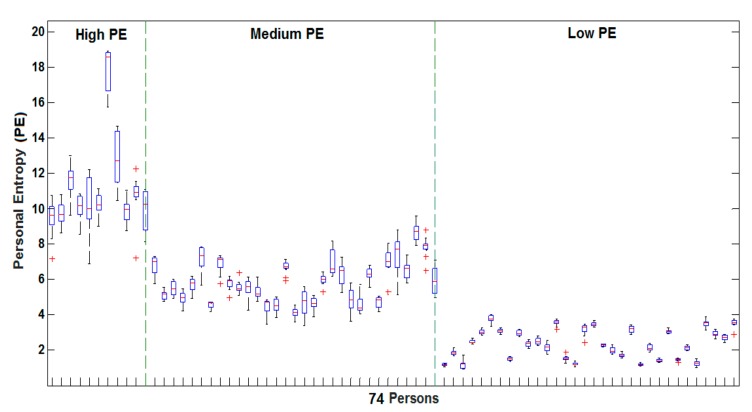
Boxplots of PE values for all 74 persons clustered into high, medium and low PE.

In Figure 7a, we observe three examples of signatures with high PE: they are the shortest and the simplest signatures, having the aspect of a flourish, and are the most variable (see Figure 8). Such signatures are considered as being “problematic” in the literature [28,43,45]. On the other hand, Figure 7c shows three examples of signatures with low PE: they are longer, the most complex and the most stable (see Figure 8). In between, there is a transition category in terms of complexity and stability, the category of medium PE (see Figure 7b and Figure 8).

### 4.2. Quality Measure of All Signature Types

**Figure 9:**
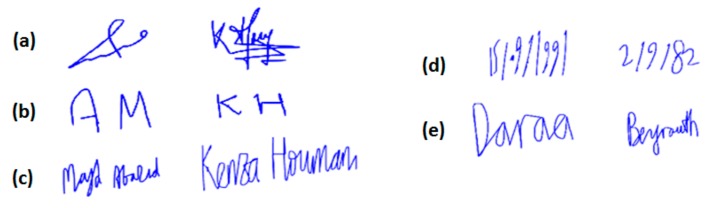
Examples of the simple signature types: (**a**) usual signature; (**b**) initials; (**c**) name-surname, (**d**) date of birth, and (**e**) place of birth. These signatures belong to persons who have authorized their publication.

Figure 9 displays two examples of each simple signature type, captured separately on the iPad: usual signature, initials, name-surname, date and place of birth.

**Figure 10:**
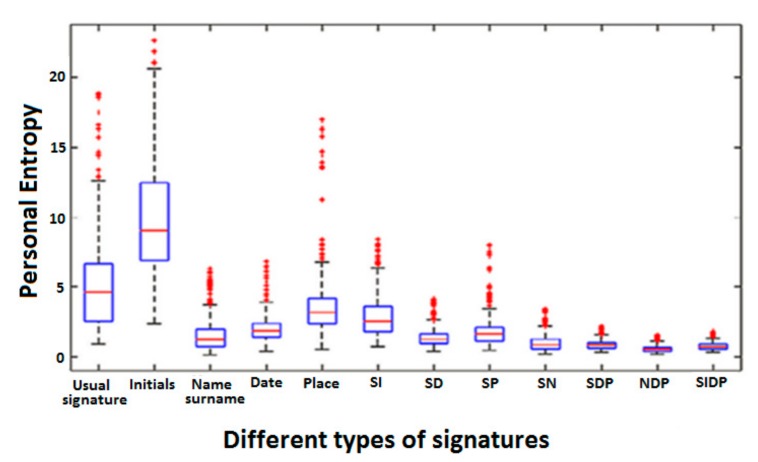
Boxplots of PE values for the 74 persons per signature type.

For each person, we compute PE values for the five simple signature types separately, and for the seven hybrid signature types: SI, SD, SP, SN, SDP, NDP and SIDP. Figure 10 presents the boxplots of the obtained PE values for the 12 signature types. 

We first notice that initials have the highest PE values. This result is coherent since initials are the most simple and variable type of signature. We also notice a strong spread out of their boxplot in Figure 10. Moreover, we observe that some initials have a comparable PE to that of usual signatures: indeed, for initials, some entropy values are below the first quartile. This can be explained by the fact that some persons appended their initials into two, three and even four letters, sometimes linking them as usually done when producing a short signature. In this case, the initials show a higher complexity and stability.

Furthermore, we notice that the more the signature is enriched (name-surname, SI, SD, SP, SN, SDP, NDP, SIDP), the lower PE becomes: the complexity of signatures is higher and variability is lower. The hybrid types SDP, NDP and SIDP are those showing the lowest PE values and the lowest variance between persons in the boxplots displayed in Figure 10. 

In the sequel, we study the relationship between information content quantified by PE and verification performance. Our objective is to identify which types of signatures are more resistant to attacks in uncontrolled mobile conditions.

### 4.3. Evaluation of the Proposed Scheme

As we have proven in former works the significant difference in verification performance between PE categories [28,40,41,42,43], we naturally adopted a methodology assessing the impact of our strategy on each PE category separately. This methodology consists in the following steps: first we computed PE values of the 74 persons considering only their usual signatures. Then, we generated three user categories based on the obtained PE values, by a Hierarchical Clustering (as explained in Section 4.1). Finally, we assessed, per user category, verification performance on usual signatures, and compared it to performance when considering the other signature types: initials, name-surname, SI, SN, NDP, SDP, and SIDP. 

For performance assessment, we considered, for each person and each signature type, the remaining 15 genuine signatures (the other 10 genuine signatures were used for PE computation) and the 10 available skilled forgeries. For each person, the HMM classifier was trained on five genuine signatures among the 15, and tested on the remaining 10 genuine instances and the 10 skilled forgeries. The same signature type is considered in the training and testing phases. 

Five random samplings were carried out on the training signatures. The false acceptance rate (FAR) and false rejection rate (FRR) are computed relying on the total number of false rejections and false acceptances obtained on all the five random samplings.

#### 4.3.1. Results on High PE Category

We analyze in this section the results obtained on the so-called “problematic” users in the literature [28,43,45], which are the main target of our strategy for enhancing signature security in uncontrolled mobile conditions.

**Figure 11:**
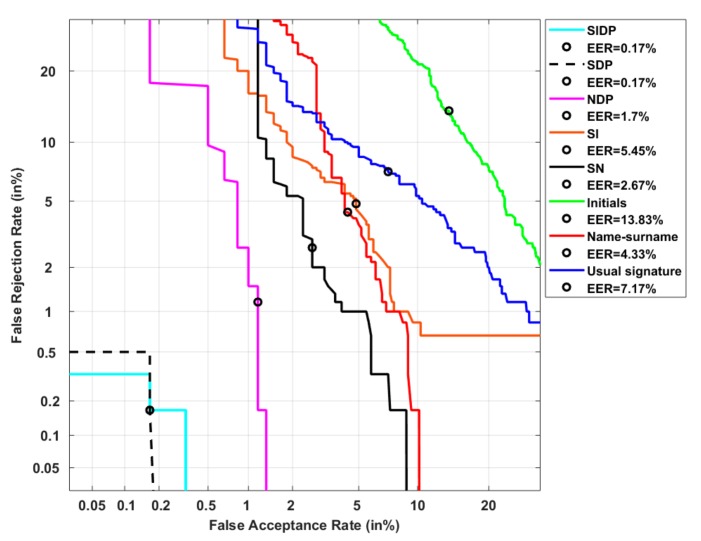
System performance on users of the highest PE category considering the 8 signature types.

**Figure 12:**
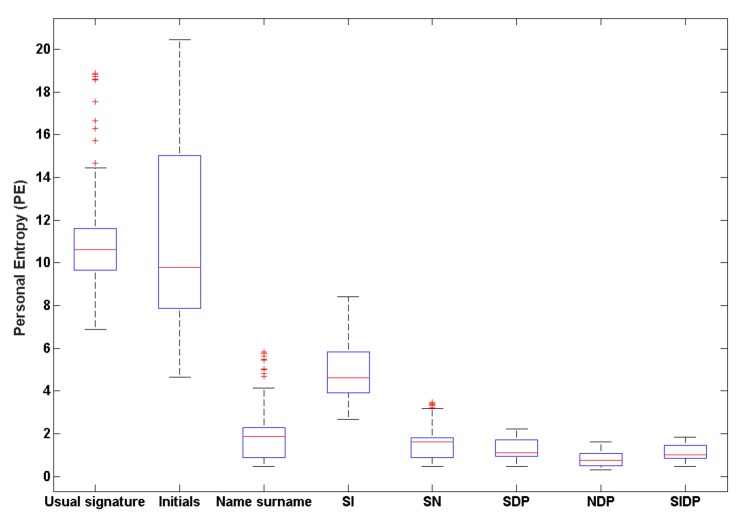
Boxplots of PE values per signature type for users of the highest PE category.

Figure 11 and Table 3 display the system performance on problematic users, those with the highest PE. The EER reaches 7.17% when considering their usual signature (see Table 3 and the blue curve in Figure 11). We first notice a significant degradation of performance when persons sign with their initials (green curve in Figure 11). A relative degradation of 93% at the EER is observed even if the usual signature is already simple and variable. This highlights the importance of the ballistic aspect of the signing process in terms of resistance to attacks. Note that the vulnerability of initials is also predictable by their very high PE values observed in Figure 12. 

Moreover, we notice a significant improvement in performance when persons sign with their name-surname (red curve in Figure 11). The FAR is in this case bounded around 10%. Also, the hybrid type SN, which combines the usual signature and name-surname, improves significantly performance (black curve in Figure 11): at the EER, the relative improvement is of 63% compared to the usual signature. This result confirms the robustness of this hybrid type to attacks, predictable by its low PE values displayed in its corresponding boxplot in Figure 12. 

Besides, adding the date and place information clearly enhances performance. Indeed, the NDP type (magenta curve in Figure 11) improves performance of 83.68% at the EER when compared to the usual signature. But the SDP type outperforms the NDP: the relative improvement is of 98% at the EER, when compared to the usual signature (see Table 3 and black dotted curve in Figure 11). Moreover, it leads to a bounded FAR at 0.2% and a bounded FRR at 0.5%. Interestingly, we notice that this could not be predicted by PE since SDP type has higher PE values than NDP (see Figure 12). This result shows that the ballistic gesture inherent to the usual signature remains more discriminant than the name-surname, when being combined to an alphanumeric information (the date) and handwriting (the place), even in the case of a very simple problematic signature.

Finally, the SIDP type does not perform significantly better than the SDP type. This may be explained by the fact that in this particular category of users, the usual signature is simple and variable, and thus close to initials in terms of information content. 

#### 4.3.2. Results on Low PE Category

**Figure 13:**
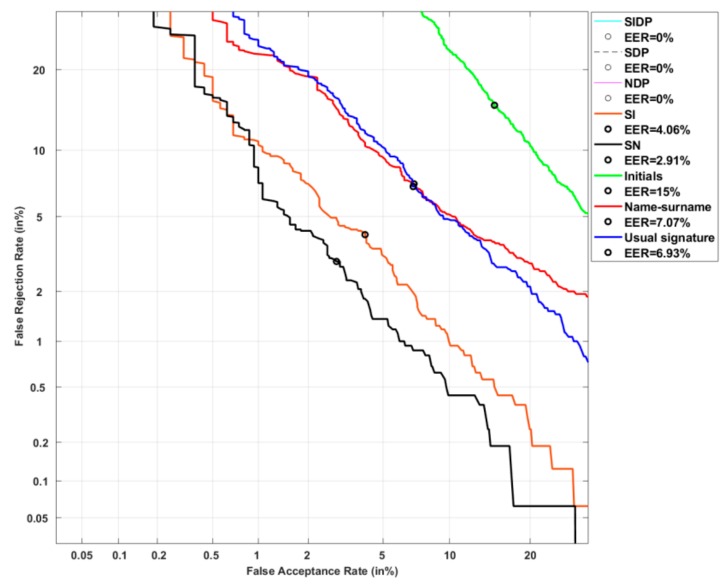
System performance on users of the lowest PE category considering the 8 signature types.

Figure 13 and Table 4 show system performance on persons with low PE, whose signatures are the most complex and stable, and the most robust to attacks. The EER reaches 6.93% (see Table 4) when considering their usual signature (blue curve in Figure 13). 

**Figure 14:**
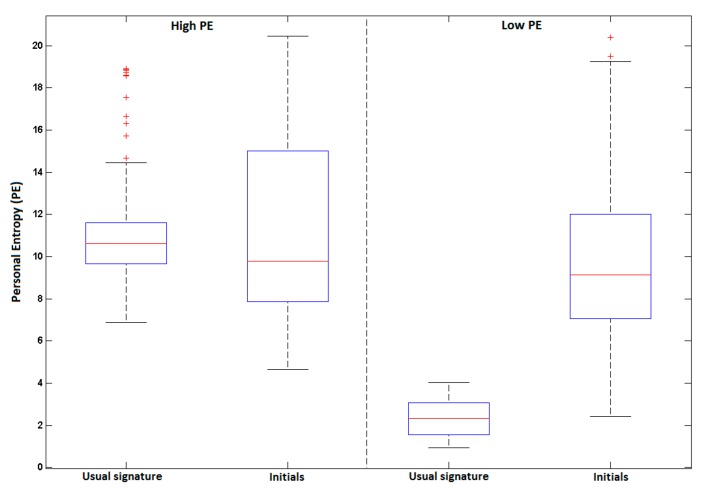
Boxplots of PE for users with highest (left) and lowest (right) PE values, considering their usual signature and initials.

Some trends observed on problematic users in the previous section are here confirmed. First, a significant degradation of 116% is obtained at the EER with initials relatively to the usual signatures. PE predicts this trend in Figure 14 (higher PE values for initials). Besides, as expected, this relative degradation of 116% is higher in the case of complex signatures of this category, compared to problematic users (relative degradation of 98% as reported in Section 4.3.1). Figure 14 shows the significant gap between initials and usual signatures for the low PE category, compared to that obtained on problematic users (high PE category). 

Moreover, the hybrid types SI, SN, NDP, SDP and SIDP outperform significantly the usual signature; we note a relative improvement of 100% at the EER for NDP, SDP and SIDP types. For this reason, the three associated DET-curves are not visible in Figure 13. This confirms again their resistance to attacks, predictable by their low PE values, as shown in Figure 14. 

**Figure 15:**
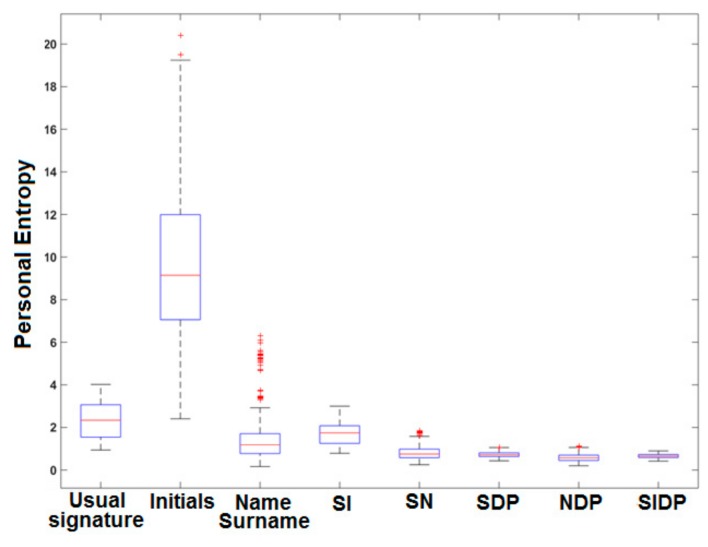
Boxplots of PE values per signature type for users of the lowest PE category.

On the other hand, some trends differ from those observed on problematic users. We notice that the name-surname type (red curve in Figure 13) gives similar performance to that of the usual signature (blue curve in Figure 13); while for problematic users, the name-surname outperforms by 40% the usual signature (see Figure 11 and Table 3). This means that in this category of persons, if we consider the name-surname as a possible signature for identity verification, although it has higher complexity (low PE in Figure 15), performance would not be improved relatively to the usual signature. 

In conclusion, this result shows on one hand that the complexity criterion is not sufficient to enhance the security of a signature. On the other hand, it highlights the importance of the ballistic process for identity verification. 

#### 4.3.3. Results on Medium PE Category

**Figure 16:**
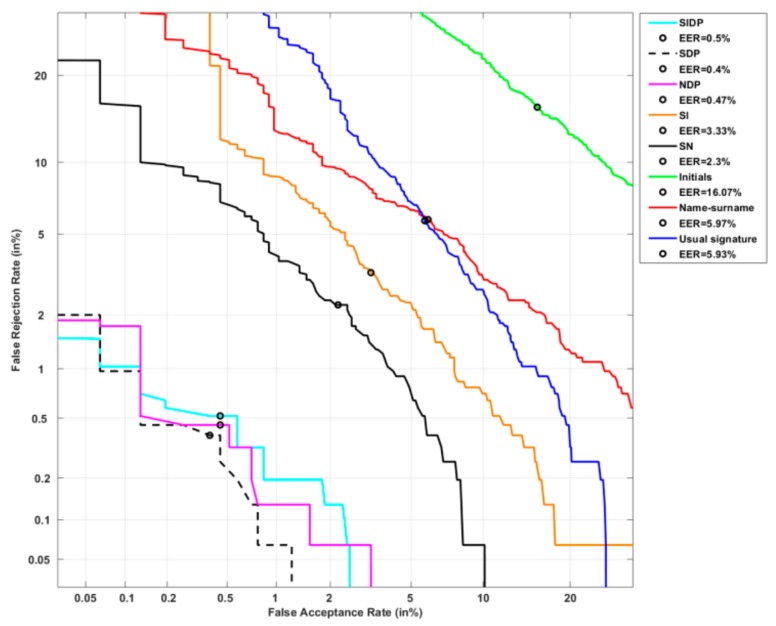
System performance on users of the medium PE category considering the 8 signature types.

Figure 16 and Table 5 display system performance on persons belonging to the category of medium PE. The obtained results on this category show an intermediate behavior compared to that observed on high and low PE categories. More precisely, the initials are the worst type in terms of performance. 

Moreover, the DET-curves corresponding to name-surname and usual signature types intersect at the EER value and get closer compared to problematic users. Also, the hybrid types including alphanumeric and handwriting information enhance significantly verification performance in uncontrolled mobile conditions. The SDP type in particular leads to a significant relative improvement of 93% at the EER compared to the usual signature; besides we note that the FAR and FRR are both bounded at 1.2% and 2%, respectively.

## 5. Discussion

In this paper, we have proposed a novel strategy for securing personal identity on a touch-screen sensor embedded in a mobile device, largely used nowadays. This strategy operates upstream from the verification system, at the sensor level, by enriching the information content of handwritten inputs. Specific additional inputs then reinforce the usual signature with alphanumeric and handwritten personal information, frequently used in public and legal usages.

We quantified information enrichment with PE measure that characterizes both signature complexity and stability. Several simple and hybrid signature types were proposed for our experimental study. 

We assessed the effectiveness of our proposal across three well-established user categories in terms of signature complexity, signature stability and verification performance. This allowed highlighting inside categories, subtle differences in terms of relative performance enhancement, depending on the signature type. This methodology allows understanding which characteristics are relevant in the signing process to reinforce the digital identity for all persons. 

Experiments were performed on 74 writers that signed on a tablet with a stylus in uncontrolled mobile conditions. Our analysis per writer category revealed a common trend to all: adding alphanumeric (date) and handwriting (place) information to the usual signature is the most powerful hybrid type in terms of verification performance. This can be explained by the fact that this hybrid type combines complementary information, and keeps the ballistic aspect of the signature, so important for identity verification. The relative improvement for all user categories is of at least 93% compared to the usual signature. 

## 6. Conclusions and Future Work

The important outcome of our study is the possibility of extending the concept of handwritten identity to other personal information than the usual signature. Actually, by combining the usual signature with alphanumeric (date) and handwritten (place) personal information, personal identity security is significantly enhanced for all persons in uncontrolled mobile conditions. Moreover, with our strategy, the concept of user categories even disappears because all persons become very robust to attacks. 

Another interesting outcome is that the complexity criterion is not sufficient to enhance the security of a signature. This is clearly observed on persons with the most complex signatures (low PE category): although the name-surname type is more complex than the usual signature, it is not more reliable in terms of resistance to attacks. This is because the usual signature conveys specific ballistic information about identity; this information can be completed by other handwritten information but cannot be removed and replaced for robust identity verification. 

The finding of combining signature, date and place for enhancing identity security is in total accordance with public and legal usages in which identity information is requested. This may facilitate the implementation of the proposed enrollment strategy at a large scale.

In future work, we envisage implementing our strategy by developing an application on different mobile devices to study the practical usage of the proposed enrollment strategy, in terms of acquisition time during enrollment, user HMM training when acquiring signature followed by date and place in one shot, accuracy in mobility, and user acceptability and comfort. This will be conducted considering challenging mobile scenarios in terms of interoperability and time variability. Also, since our study demonstrates that augmenting the usual signature with alphanumeric and handwritten personal information enhances significantly verification performance, it would be interesting to study the impact of reducing the number of enrollment inputs. Furthermore, we will investigate the effectiveness of our strategy in terms of relative performance improvement when confronted to other classifiers. 

## Figures and Tables

**Table 1:** Performance of our HMM-based ASV system on several online signature databases acquired in office-like (Wacom device) and mobile scenarios (touch-screen sensors), considering skilled forgeries.

Databases	Evaluation Campaigns	Year	Devices	Users	EER (in %)
DS2-382 [15]	BSEC’2009	2012	Wacom tablet	382	4.47
DS3-382 [15]	BSEC’2009	2012	PDA (stylus)	382	11.27
DS2-382 [16]	ESRA’2011	2011	Wacom tablet	382	2.73–4.04
DS3-382 [16]	ESRA’2011	2011	PDA (stylus)	382	8.13–10.92
DS3-210 [27]	-	2010	PDA (stylus)	210	9.95
PDA-64 [27]	-	2010	PDA (stylus)	64	16.02
iPad-74 [46]	-	2019	iPad (stylus)	74	7.04
iPhone-74 [46]	-	2019	iPhone (stylus)	74	4.95
BIOMET [47]	-	2007	Wacom tablet	84	2.33
PHILIPS [47]	-	2007	Digitizing tablet	51	3.25
SVC2004 [47]	-	2007	Digitizing tablet	40	4.83
MCYT-100 [47]	-	2007	Wacom tablet	100	3.37
MCYT-330 [48]	-	2009	Wacom tablet	330	3.91

**Table 2:** Performance of ASV systems of the literature on several online signature databases acquired in mobile scenarios with touch-screen sensors, considering skilled forgeries.

Databases	Year	Users	Sensor	EER (in %)
ATVS-DooDB [32]	2013	100	HTC Touch HD (Pseudo-signatures)	Finger: 26.9
Blanco-Gonzalo et al. [31]	2013	43	Asus Eee PC Touch (stylus) Samsung Gal. Note (stylus/finger) BlackBerry Playbook (finger) Apple Ipad2 (finger) Samsung Gal. Tab (finger)	-
e-Biosign [37]	2016	65	Samsung ATIV7 Samsung Gal. Note	Stylus: 7.9 Finger: 22.1 Stylus: 10.7 Finger: 26.4
Zareen and Jabin [38]	2016	25	Samsung Gal. Note	-
MOBISIG [36]	2018	83	Nexus 9 tablet capacitive (Finger-drawn pseudo-signatures)	Personalized vs. global threshold: 8.56% vs. 25.45%
Nam et al. [39]	2018	20	Samsung Gal. S3	Finger: 4.4%

**Table 3:** System performance on users of the highest PE category in terms of EER.

Type of Signatures	EER
Usual signature	7.17%
Initials	13.83%
Name-surname	4.33%
SN	2.67%
SI	5.45%
NDP	1.7%
SDP	0.17%
SIDP	0.17%

**Table 4:** System performance on users of the lowest PE category in terms of EER.

Type of Signatures	EER
Usual signature	6.93%
Initials	15%
Name-surname	7.07%
SN	2.91%
SI	4.06%
NDP	0%
SDP	0%
SIDP	0%

**Table 5:** System performance on users of the medium PE category in terms of EER.

Type of Signatures	EER
Usual signature	5.93%
Initials	16.07%
Name-surname	5.97%
SN	2.3%
SI	3.33%
NDP	0.47%
SDP	0.4%
SIDP	0.5%

## Appendix A

**Table A1:** The 19 dynamic features extracted point-wise on all signature types.

	N°	Feature Name
Gesture related features	1–2	Normalized coordinates (*x(t)−x_g_, y(t)−y_g_*) relative to the gravity center (*x_g_, y_g_*) of the signature
	3–4	Speed in *x* and *y*
	5	Absolute speed
	6	Ratio of the minimum over the maximum speed on a window of 5 points
	7–8	Acceleration in *x* and *y*
	9	Absolute acceleration
	10	Tangential acceleration
Local shape related features	11	Angle α between the absolute speed vector and the *x* axis
	12–13	Sine(α) and Cosine(α)
	14	Variation of the α angle: Φ
	15–16	Sine(Φ) and Cosine(Φ)
	17	Log(1 + r) where r is the curvature radius of the signature at the present point
	18	Length to width ratio on windows of size of 5 points
	19	Length to width ratio on windows of size of 7 points

For computing the derivatives of a parameter $x\left(t\right)$*,* we used the regression equation as follows:

$$x^{\prime }\left(t\right)=reg\left(x\left(t\right),Z\right)=\frac{\sum_{z=1}^{Z}z*\left(x\left(t+z\right)-x\left(t-z\right)\right)}{2\;\sum_{z=1}^{Z}z^{2}}$$

where z = 2, in order to obtain soft derivative curves.

Accordingly,

- Speed in *x* and *y* (N° 3–4 in Table A1):
  $$v_{x}\left(t\right)=reg\left(x\left(t\right),2\right)\;\;\;\;\;\;\;\;\;\;v_{y}\left(t\right)=reg\left(y\left(t\right),2\right)$$
- The absolute speed (N° 5 in Table A1):
  $$v\left(t\right)=\sqrt{v_{x}^{2\;}\left(t\right)+v_{y}^{2\;}\left(t\right)}$$
- Acceleration in x and y (N° 7–8 in Table A1):
  $$\;a_{x}\left(t\right)=reg\left(v_{x}\left(t\right),2\right)\;\;\;\;\;\;\;\;\;\;a_{y}\left(t\right)=reg\left(v_{y}\left(t\right),2\right)$$
- The absolute acceleration (N° 9 in Table A1):
  $$a\left(t\right)=\sqrt{a_{x}^{2\;}\left(t\right)+a_{y}^{2\;}\left(t\right)}$$
- The tangential acceleration (N° 10 in Table A1):
  $$a_{t}\left(t\right)=reg\left(v\left(t\right),2\right)$$
- Angle *α* between the absolute speed vector and the x axis (N° 11 in Table A1):
  $$\alpha \left(t\right)=arcsin\left(\frac{v_{y}\left(t\right)}{v\left(t\right)}\right)$$
- Sine and cosine of angle *α* (N° 12–13 in Table A1):
  $$Sin\left(\alpha \left(t\right)\right)=\left(\frac{v_{y}\left(t\right)}{v\left(t\right)}\right)\;\;\;\;\;\;\;\;\;\;\mathrm{Cos}\left(\alpha \left(t\right)\right)=\left(\frac{v_{x}\left(t\right)}{v\left(t\right)}\right)$$
- Variation ϕ of the angle α angle (N° 14 in Table A1):
  $$\phi \left(t\right)=reg\left(\alpha \left(t\right),2\right)$$
- Sine and cosine of angle ϕ (N° 15–16 in Table A1):
  $$sin\left(\phi \left(t\right)\right)\;cos\left(\phi \left(t\right)\right)$$
- $\log\left(1+r\left(t\right)\right)$ (N° 17 in Table A1), where r is the curvature radius of the signature at the present point *t*:
  $$lr\left(t\right)=\log\left(1+r\left(t\right)\right)=log\left(1+\frac{v_{t}\left(t\right)}{\phi \left(t\right)}\right)$$
- Length to width ratio on windows of size of 5 points centered on the current point t (N° 18 in Table A1).
- Length to width ratio on windows of size of 7 points centered on the current point t (N° 19 in Table A1).
